# Joint Associations of Food Groups with All-Cause and Cause-Specific Mortality in the Mr. OS and Ms. OS Study: A Prospective Cohort

**DOI:** 10.3390/nu14193915

**Published:** 2022-09-21

**Authors:** Jingli Yang, Aimin Yang, Suey Yeung, Jean Woo, Kenneth Lo

**Affiliations:** 1 College of Earth and Environmental Sciences, Lanzhou University, Lanzhou 730000, China; 2 School of Public Health and Social Work, Queensland University of Technology, Brisbane 4059, Australia; 3 Department of Medicine and Therapeutics, The Chinese University of Hong Kong, Shatin, New Territories, Hong Kong SAR, China; 4 Department of Applied Biology and Chemical Technology, The Hong Kong Polytechnic University, 11 Yuk Choi Road, Hung Hom, Kowloon, Hong Kong SAR, China; 5 Research Institute for Smart Ageing, The Hong Kong Polytechnic University, Hong Kong SAR, China

**Keywords:** food groups, mortality, cardiovascular, cancer, prospective cohort

## Abstract

Despite continuous growth in dietary pattern research, the relative importance of each dietary component in the overall pattern and their joint effects on mortality risk have not been examined adequately. We explored the individual and joint associations of multiple food groups with all-cause and cause-specific mortality (cardiovascular disease (CVD) or cancer), by analyzing data from a cohort of 3995 Hong Kong Chinese older adults in the Mr. Osteoporosis (OS) and Ms. OS Study. Cox proportional hazards models were used to examine the associations of food groups with mortality risk. The individual and joint contribution of food groups to mortality risk has been quantified by a machine learning approach, i.e., the Quantile G-Computation. When comparing the highest with the lowest quartile of intake, dark green and leafy vegetables (hazard ratio (HR) = 0.82, 95% confidence interval (CI) = 0.70 to 0.96, *P*_trend_ = 0.049), fruit (HR = 0.79, 95% CI = 0.68 to 0.93, *P*_trend_ = 0.006), legumes (HR = 0.75, 95% CI = 0.63 to 0.87, *P*_trend_ = 0.052), mushroom and fungi (HR = 0.76, 95% CI = 0.65 to 0.88, *P*_trend_ = 0.023), soy and soy products (HR = 0.77, 95% CI = 0.66 to 0.90, *P*_trend_ = 0.143), and whole grains (HR = 0.76, 95% CI = 0.65 to 0.89, *P*_trend_ = 0.008) were inversely associated with all-cause mortality. Legume intake was associated with a lower risk of CVD mortality, while fruit, nuts, soy and soy products were associated with a lower risk of cancer mortality. From the Quantile G-Computation, whole grains, legumes, fruits, mushroom and fungi, soy and soy products had a higher relative weighting on mortality risk, and the joint effect of food groups was inversely associated with the mortality risk due to all-causes (HR = 0.39, 95% CI = 0.27 to 0.55), CVD (HR = 0.78, 95% CI = 0.67 to 0.91), and cancer (HR = 0.31, 95% CI = 0.15 to 0.65). From a sex-stratified analysis, most associations between food groups (whole grains, legumes, fruits, mushroom and fungi, soy and soy products) and mortality risk remained significant among men. In conclusion, whole grains, legumes, fruits, mushroom and fungi, soy and soy products were the main contributors to a reduction in mortality risk, and their joint effects were stronger than individual food groups. Moreover, the sex-specific association of sweets and desserts with cancer mortality may be worth further investigation.

## 1. Introduction

Maintaining a healthy diet is a major lifestyle factor in preventing multiple chronic diseases, including diabetes, cardiovascular diseases (CVD), and cancer [1]. While multiple studies have evaluated the relationships between individual nutrients and health, emerging evidence has suggested dietary patterns to explain the interactions between different foods and food components [2]. There are some common features of healthy dietary patterns, including the consumption of wholegrain cereals, fruits, and vegetables, that are recommended by the World Health Organization Global Strategy on Diet, Physical Activity, and Health [3], and cholesterol-lowering foods, namely oats, barley, nuts, and plant protein foods (e.g., soy and other legumes) [4]. In addition, a higher adherence to plant-based diets, especially those rich in a variety of plant foods (whole grains, fruits, vegetables, nuts, legumes, and vegetable oils), may be associated with decreased weight gain and lower adiposity in prospective cohorts among the general population [5,6].

Despite continuous growth in dietary pattern research, there are some limitations in the current statistical approaches to analyzing the association between dietary patterns and health outcomes. First, the health effects of the specific dietary component could be diluted within the total dietary pattern [7], while the relative importance of each component in the overall pattern is seldom verified by a quantitative approach [8]. Second, the associations between healthy dietary patterns and health outcomes are mainly examined by conventional regression models, such as logistic regression and Cox proportional hazards regression. Although regression analysis can adjust for multiple confounders, these models have not accounted for the synergy of dietary factors on health outcomes [9].

To address the knowledge gap, sophisticated statistical methods that can investigate the single and joint effects of dietary components on health in a prospective study are necessary. A few studies in recent years have explored the interaction effects of food groups on cardiovascular health and pregnancy outcomes [10,11]. In the present study, we aim to supplement the traditional analytic methods of nutritional epidemiology using Quantile G-Computation, a machine-learning-based approach that can examine the influence of dietary factors on disease outcomes as a mixture of exposures. The findings will help to reveal new diet–disease relationships in a sex-specific manner and identify beneficial or detrimental food groups that are specific to different populations.

## 2. Materials and Methods

### 2.1. Study Design and Population

We analyzed data from a prospective cohort, the Mr. Osteoporosis and Ms. Osteoporosis Study in Hong Kong (Mr. OS and Ms. OS study), and evaluated the associations of food groups with all-cause and cause-specific mortality [12]. The Mr. OS and Ms. OS study is a prospective cohort study that recruited 4000 community-living Hong Kong Chinese men and women aged at least 65 years in the period 2001–2003 [12]. The target was to recruit a stratified sample that equally assigned the cohort into three age groups (65–69, 70–74, and ≥75 years). Compared with the general elderly population in Hong Kong in this age group, the participants had higher educational levels (9.8% vs. 3.8% with tertiary education), a higher proportion that were married (70.7% vs. 59.9%) and a slightly lower proportion of those living alone (10.8% vs. 11.3%) [13]. The Mr. OS and Ms. OS study was conducted according to the guidelines laid down in the Declaration of Helsinki, and all procedures involving human subjects were approved by the Clinical Research Ethics Committee of the Chinese University of Hong Kong (CRE: 2003.102). This study is reported as per the Strengthening the Reporting of Observational Studies in Epidemiology (STROBE) guideline for nutritional epidemiology (Table S1).

### 2.2. Dietary Assessment

A dietary assessment was conducted at a baseline level using a validated food frequency questionnaire (FFQ) (OS FFQ hereafter) with 280 food items validated against 24 h dietary recalls [14,15]. Each participant was asked to respond to the FFQ by trained personnel in a face-to-face interview, answering how often they consumed each food item each day or each week, and the size of each portion, over the past year. A pictorial catalogue with individual food portions was provided as a guide. The daily amount of consumption of 34 food groups (in grams/day) including fruits, vegetables, whole grains, refined grain, eggs, milk and milk products, poultry, and red and processed meat, etc., was calculated. The consumption of each food group in quartiles (Q) is set out in Table S2.

### 2.3. Ascertainment of Mortality Outcomes

Data on the mortality statistics of all participants were obtained from the Death Registry of the Department of Health of Hong Kong and collected through to 31 March 2017 [13]. Causes of death were defined by the International Classification of Disease (ICD) version 10 codes, and classified as all-cause, CVD-specific (I00–I99) and cancer-specific (C00–D49) mortality [14].

### 2.4. Covariates

Demographic, lifestyle, and health information were all collected at a baseline level, including the education level (secondary school or below vs. post-secondary education), smoking habits (never, former or current smoker), alcohol consumption (≥12 drinks of beer, wine, including Chinese wine, or liquor over the previous year), physical activity, dietary intake, and medical history (diabetes, hypertension, stroke, heart attack, angina, congestive heart failure or cancer). Physical activity levels were evaluated using the Physical Activity Scale for the Elderly (PASE), and a higher PASE score represented a higher intensity of physical activity in which the older adults engaged [16]. Body weight was measured using the Physician Balance Beam Scale (Healthometer, McCook, NE, USA) to the nearest 0.1 kg, with participants wearing a light gown. Height was measured to the nearest 0.1 cm using the Holtain Harpenden Stadiometer (Holtain Ltd., Pembrokeshire Wales, UK), which was used to compute body mass index (BMI).

### 2.5. Statistical Analysis

Baseline characteristics were stratified by sex (men or women) using means with standard deviations for continuous variables and frequencies with percentages for categorical variables. To compare baseline characteristics, the Mann–Whitney *U* test was used for continuous variables and χ^2^ test for categorical variables. We performed three stages of data analyses to identify the individual and joint associations in food groups that had the most substantial influence on mortality risk (Figure S1). Since 10 out of 34 (29.4%) food groups had ≥50% participants with zero intake and may have complicated the result interpretation, we included 24 (70.6%) food groups that at least half of the study population had consumed for further analysis (Table S2).

In stage 1, we used the elastic net penalty regression to identify the most important food groups that were associated with mortality outcomes (all-cause, CVD, and cancer) [17]. The elastic net model can perform selection, and enable the inclusion of collinear predictors through combining the least absolute shrinkage and the selection operator and ridge. We performed a 10-fold cross validation to acquire the minimum mean squared error (MSE) for an unbiased and robust estimate of prediction accuracy [18]. A set of elastic net coefficients (β_EN_) were estimated by minimizing the MSE. The β_EN_ represented the change in mean outcome variables per increment of each food group. If the absolute value of β_EN_ had not shrunk to zero with a minimum MSE, the related food group was selected [19]. We performed elastic net penalty regressions separately for each mortality outcome to select the set of food groups.

In stage 2, participants were categorized into Q according to the consumption of each food group, with the lowest Q serving as the reference group. Cox proportional hazards models were used to estimate hazard ratios (HRs) and 95% confidence intervals (CIs) for the associations between the food group consumption Q and mortality outcomes. The Cox proportional hazards models were adjusted for sex (men vs. women), age, dietary energy, BMI, physical activity (all continuous variables), medical history (yes or no), smoking habits (never, former or current smokers), alcohol drinking (yes or no), and education level (post-secondary education vs. secondary school or below). We also applied subgroup analyses by sex. Trend analysis was performed by assigning median values to each food group quartile and treating it as a continuous variable in the regression model [20].

In stage 3, we applied the Quantile G-Computation, a machine-learning method, to evaluate the importance of each food group and their joint effects on the risk of mortality [21,22]. The qgcomp.cox.noboot function in the R qgcomp package was used to estimate the exposure effects, which firstly categorizes all food groups into Q, then assigns each food group with a positive or negative weight on their relationship with the outcomes. If the included food groups were associated with mortality in different directions, the positive or negative weights were interpreted as the percentage of exposure effects that had a positive or inverse association with mortality outcomes, with the positive and negative weights each adding up to one. In addition, a qgcomp index was computed based on the variable-specific coefficients for each included food group, and the association between the index and the risk of mortality was examined [21,22]. In other words, the qgcomp index summarized the joint effect of increasing one quartile of each food group with a negative (positive) weight with the cause-specific mortality risk, and the overall effect was presented as HR (95% CI). The Quantile G-Computation was adjusted for sex, age, dietary energy, body mass index, physical activity, medical history, smoking habits, alcohol drinking, and education level. We repeated all analyses in both men and women.

Statistical tests were two-sided and *p* < 0.05 was considered statistically significant. All statistical analyses were conducted with R 4.1.2 (R Foundation for Statistical Computing, Vienna, Austria).

## 3. Results

### 3.1. Characteristics of the Study Participants

After excluding five people with missing information on dietary intake or covariates, 3995 (99.9%) participants were included in the analyses. During a median (interquartile range) of 13.75 (11.60 to 14.51) years of follow-up, we documented 1370 cases (34.3%) of all-cause mortality, 317 cases (7.9%) of CVD mortality, 469 cases (11.7%) of cancer mortality. The mortality rates due to all-causes, CVD or cancer were 27.73 (95% CI = 26.34 to 29.12), 6.36 (95% CI = 5.60 to 7.12), and 9.49 (95% CI = 8.58 to 10.40) events per 1000 person-years. The baseline characteristics of the participants in this study are presented in Table 1. As shown by the Mann–Whitney U test, men tended to have, on average, higher dietary energy, lower BMI, and higher physical activity compared to women. As demonstrated by the chi-square test, men were more prevalent in the categories of suffering from a stroke, angina, being a smoker or alcohol drinker, and having post-secondary education compared to women. Except for cruciferous vegetables, legumes, and starchy vegetables, there were sex-specific differences between men and women in the dietary consumption of food groups (Table S3).

### 3.2. Food Group Selection Using Elastic Net Regression Model

Elastic net regression models were performed in 24 food groups to identify the associations with mortality risk. Three sets of food groups with non-zero coefficients of β_EN_ were selected according to each mortality outcome (all-cause, CVD or cancer) (Figures S2–S4). We selected 10 food groups which were associated with an all-cause mortality risk (β_EN_ varied from −0.0033 to 0.0014) (Table S4). For CVD mortality, 3 food groups were selected (β_EN_ < −0.0001 for Legumes, starchy vegetables, and tomatoes) (Table S4). For cancer mortality, 12 food groups were selected (β_EN_ varied from −0.0009 to 0.0038) (Table S4).

### 3.3. Food Groups and All-Cause Mortality

Table 2 has presented the associations between food groups and all-cause mortality in the single-food group model. When comparing the extreme Q (Q4 versus Q1), the reduced risk of all-cause mortality was observed for dark green and leafy vegetables (HR = 0.82, 95% CI = 0.70 to 0.96, *P*_trend_ = 0.049), fruit (HR = 0.79, 95% CI = 0.68 to 0.93, *P*_trend_ = 0.006), legumes (HR = 0.75, 95% CI = 0.63 to 0.87, *P*_trend_ = 0.052), mushroom and fungi (HR = 0.76, 95% CI = 0.65 to 0.88, *P*_trend_ = 0.023), soy and soy products (HR = 0.77, 95% CI = 0.66 to 0.90, *P*_trend_ = 0.143), and whole grains (HR = 0.76, 95% CI = 0.65 to 0.89, *P*_trend_ = 0.008).

**Figure 1:**
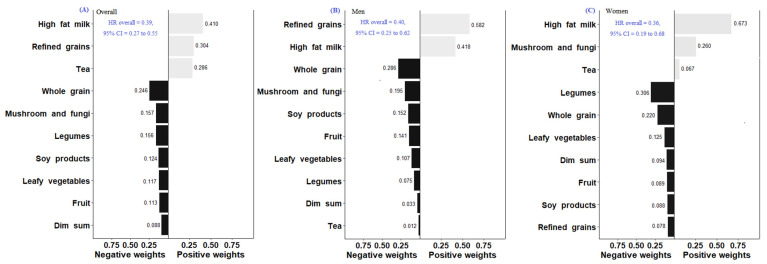
Weights representing the proportion of the positive or negative partial effects of each food group on all-mortality risk in the Quantile G-Computation Overall (**A**), Men (**B**), and Women (**C**). Model adjusted for sex, age, dietary energy, body mass index, physical activity, systolic blood pressure, medical history (diabetes, hypertension, stroke, heart attack, angina, congestive heart failure or cancer), smoking habits, alcohol drinking, and education level.

In the Quantile G-Computation of ten food groups (Figure 1), high-fat milk and milk products had the highest relative weighting (weighted at 0.410) among three food groups with positive weights. Whole grains had the highest negative weighting (weighted at 0.246) among seven food groups with negative weights. The qgcomp index has also inversely associated with the risk of all-cause mortality (HR = 0.39, 95% CI = 0.27 to 0.55).

### 3.4. Food Groups and Cause-Specific Mortality

**Figure 2:**
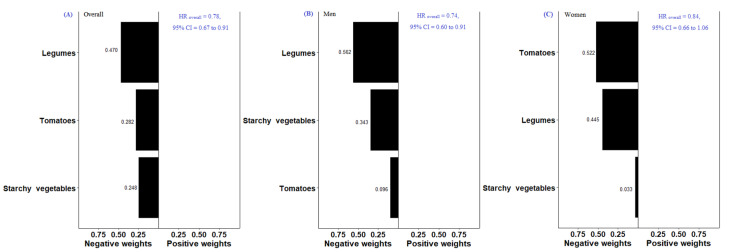
Weights representing the proportion of the positive or negative partial effect for each food group on CVD-mortality risk in the Quantile G-Computation Overall (**A**), Men (**B**), and Women (**C**). Model adjusted for sex, age, dietary energy, body mass index, physical activity, systolic blood pressure, medical history (diabetes, hypertension, stroke, heart attack, angina, congestive heart failure or cancer), smoking habits, alcohol drinking, and education level.

Table 3 presents the association between food groups and CVD mortality in the single-food group model. When comparing the food intake at Q4 versus Q1, legume intake was associated with a lower risk of CVD mortality overall (HR = 0.64, 95% CI = 0.45 to 0.91, *P*_trend_ = 0.065). However, neither the highest quartile of intake for starchy vegetables (HR = 0.81, 95% CI = 0.59 to 1.12, *P*_trend_ = 0.085) nor tomatoes (HR = 0.74, 95% CI = 0.52 to 1.04, *P*_trend_ = 0.039) were associated with the risk of CVD mortality. In Quantile G-Computation of three food groups (Figure 2), all three selected food groups had negative weights on the outcome, implying an inverse association with CVD mortality. Legumes had the highest negative weight on the association with CVD mortality (weighted at 0.470). The qgcomp index was also inversely associated with the risk of CVD mortality (HR = 0.78, 95% CI = 0.67 to 0.91).

**Figure 3:**
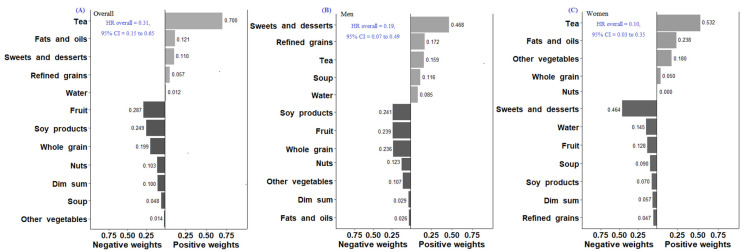
Weights representing the proportion of the positive or negative partial effects of each food group on cancer-mortality risk in the Quantile G-Computation Overall (**A**), Men (**B**), and Women (**C**). Model adjusted for sex, age, dietary energy, body mass index, physical activity, systolic blood pressure, medical history (diabetes, hypertension, stroke, heart attack, angina, congestive heart failure or cancer), smoking habits, alcohol drinking, and education level.

When comparing the food intake at Q4 versus Q1, fruit (HR = 0.68, 95% CI = 0.51 to 0.89, *P*_trend_ = 0.002), nuts (HR = 0.72, 95% CI = 0.55 to 0.94, *P*_trend_ = 0.550), soy and soy products (HR = 0.72, 95% CI = 0.54 to 0.95, *P*_trend_ = 0.051) were associated with a lower risk of cancer mortality (Table 4). In the Quantile G-Computation of twelve food groups (Figure 3), tea had the highest relative weighting (weighted at 0.700) among five food groups with positive weights. Fruit had the highest negative weighting (weighted at 0.287) among seven food groups. The qgcomp index was also inversely associated with the risk of cancer mortality (HR = 0.31, 95% CI = 0.15 to 0.65).

### 3.5. Sex-Stratified Subgroup Analysis

In the single-food group model, the associations between food groups and all-cause mortality remained significant when stratified by sex, except for fruit (HR = 0.84, 95% CI = 0.63 to 1.10, *P*_trend_ = 0.008) and mushroom and fungi (HR = 0.87, 95% CI = 0.67 to 1.13, *P*_trend_ = 0.023) among women (Table 2 and Table S5). When comparing the extreme Q, legume intake was associated with a lower risk of CVD mortality among men (HR = 0.62, 95% CI = 0.41 to 0.94, *P*_trend_ = 0.094) (Table 3 and Table S6). Similar to all participants, the association between the intake of fruit (HR = 0.62, 95% CI = 0.44 to 0.88, *P*_trend_ = 0.001), nuts (HR = 0.67, 95% CI = 0.48 to 0.94, *P*_trend_ = 0.776), soy and soy products (HR = 0.69, 95% CI = 0.49 to 0.98, *P*_trend_ = 0.196) was inversely associated with cancer mortality among men, and tea (HR = 2.16, 95% CI = 1.38 to 3.37, *P*_trend_ = 0.013) was positively associated with cancer mortality among women (Table 4 and Table S7).

In the Quantile G-Computation for all-cause mortality (Figure 1), refined grains had the highest relative weighting (weighted at 0.582) among two food groups with positive weights. Whole grains had the highest negative weighting (weighted at 0.286) among eight food groups with negative weights among men. For women, high-fat milk and milk products had the highest relative weighting (weighted at 0.673) among three food groups with positive effects on all-cause mortality. Legumes had the highest negative weighting (weighted at 0.306) among seven food groups with negative effects. We found that the qgcomp index was inversely associated with the risk of all-cause mortality among men (HR = 0.40, 95% CI = 0.25 to 0.62) and women (HR = 0.36, 95% CI = 0.19 to 0.68). For CVD mortality (Figure 2), the foods with the highest negative weight on the association were legumes among men (weighted at 0.562) and tomatoes among women (weighted at 0.522) The qgcomp index was inversely associated with the risk of CVD mortality among men (HR = 0.74, 95% CI = 0.61 to 0.91) but not women (HR = 0.84, 95% CI = 0.66 to 1.06). For cancer mortality (Figure 3), sweets and desserts had the highest relative weighting (weighted at 0.468) among five food groups with positive weights. Soy and soy products had the highest negative weighting (weighted at 0.241) among seven food groups among men. For women, tea had the highest relative weighting (weighted at 0.532) among four food groups with positive weights. Sweets and desserts had the highest negative weighting (weighted at 0.464) among eight food groups. The qgcomp index was also inversely associated with the risk of cancer mortality among men (HR = 0.19, 95% CI = 0.07 to 0.49) and women (HR = 0.10, 95% CI = 0.03 to 0.35).

## 4. Discussion

In this prospective cohort analysis of ~4000 Hong Kong older adults, we have examined the individual and joint effects of food groups on cause-specific mortality risks with a combination of conventional and advanced statistical methodologies. Whole grains, legumes, mushroom and fungi, and soy and soy products were the main identified food groups associated with a lower risk of all-cause and CVD-cause mortality, moreover, fruits, and soy and soy products were the more important food groups associated with a lower risk of death due to cancer. Furthermore, the overall effects of all food groups included in this study were more strongly associated with a lower mortality risk than individual food groups regardless of outcomes. Our analysis has demonstrated how the importance of each food group can be evaluated in the totality of a healthy dietary pattern.

### 4.1. Comparison with Previous Literature

The food groups that are the main contributors to the risk of mortality, as identified by Quantile G-Computation, were generally consistent with the evidence from previous literature. High-fat milk and milk products and refined grains provided the highest contribution to the all-cause mortality risk, which can be explained by their high contents of saturated fat and their high glycemic index. While the association between saturated fat and cause-specific mortality was supported by a meta-analysis of prospective cohorts [23], the certainty of evidence on the association between glycemic index and mortality requires improvement [24].

For foods that have a negative weight on mortality risk (whole grains, fruits, legumes, mushroom and fungi, and soy and soy products), these are rich sources of dietary fiber, which may improve health through multiple biological mechanisms, such as triggering satiety cues, delaying gastric emptying and prolonging nutrient absorption, improving the blood lipid profile, and protecting against oxidative stress [25]. Furthermore, dietary fiber may serve as the prebiotic substrates for gut microbiota to ferment and produce bioactive metabolites that can benefit cardiometabolic health [25]. For example, dietary fiber can be fermented by gut microbiota into various types of short-chain fatty acids (SCFAs) [26], mainly acetate, propionate, and butyrate, which are involved in many physiological functions [27], and enhance gut microbiota diversity [28]. The literature has also identified specific SCFA-producing bacteria, such as *Faecalibacterium* that ferment pectin from apples, oranges, and carrots, and fructans from onions, bananas, and garlics [29].

### 4.2. Sex- and Population-Specific Associations of Food Groups with Mortality Risk

In the present study, we not only identified general classes of foods that are beneficial for long-term health, but also observed several sex- and population-specific associations of food groups with mortality risk. First, sweets and desserts had a positive weight on the risk of cancer mortality in men but had a negative weight among women. Although sex-specific associations were not examined, the analysis of two prospective cohorts in Sweden has found an inverse significant association between intakes of treats (mainly cookies, cakes, pies, and buns) and all-cause mortality [30]. The authors postulated that low treat consumption might reflect fewer social connections [31], hence a higher risk of mortality [32]. Furthermore, in another analysis of Mr. OS and Ms. OS Study data, participants with a relatively high sugar intake were more likely to have higher educational levels, a lower prevalence of diabetes and hypertension, a higher intake of vitamin D, calcium and milk, and less fat consumption than their counterparts [33]. The way in which sugar may influence mortality risk according to sex and dietary source (liquid or solid) requires further investigation, especially among older adults.

Second, we have demonstrated that the joint HRs of legumes, tomatoes, and starchy vegetables (computed as the qgcomp index) was inversely associated with the risk of CVD mortality significantly overall (HR = 0.78) and among men (HR = 0.74). Legumes are a rich source of flavonoids, vitamin E and α-linolenic acid, which have a significant impact in preventing CVD [34]. Meanwhile, tomatoes are a source of lycopene, and meta-analyses suggest that it may have positive effects on blood lipids, blood pressure, and endothelial function [35], but the relationship with CVD prevention is still inconclusive [36]. For starchy vegetables, their role as part of a healthy dietary pattern is still under debate, because of its diversity in nutritional composition across populations [37]. It is notable that the individual effects of each food group was not significant in the Cox regression model. As demonstrated by another analysis of the Mr. OS and Ms. OS Study, the adherence to a cholesterol-lowering plant-based dietary pattern (the Portfolio Diet) was associated with a 28% lower risk of all-cause and cancer mortality [12], which echoes the perspective that nutrients may provide summative health benefits in the context of general dietary patterns.

Third, tea consumption was positively associated with cancer mortality, particularly for women. Tea has been viewed as an anti-cancer agent by multiple pathways, such as promoting antioxidant activity, inhibiting NF-κB and AP-1, regulating cell cycle, inhibiting receptor tyrosine kinase pathways, controlling epigenetic modifications, and modulating the immune system [38]. However, a recent meta-analysis has examined the association between tea consumption and 26 cancer sites from 113 individual studies, and has found no consistent associations for most comparisons except for the association between lymphoid neoplasms and green tea [39]. The authors suggested that a well-defined exposure of tea drinking by considering the degree of fermentation, the sources, and the water temperature of tea, will help to clarify the inconsistency in research findings [39]. In the present study, tea consumption was calculated from the total intake of green and black tea, and the outcome was defined as death from any cancer. Although our data has not provided a detailed breakdown of tea, the Quantile G-Computation provided important information on the relative weighting of tea in cancer mortality among older adults, generating a hypothesis for further studies.

### 4.3. Advantages of the Present Statistical Approach

In the present study, food groups that were associated with each mortality outcome were selected by elastic net penalty regression, followed by Quantile G-Computation to assess how the joint increment of included food groups might influence mortality risk. Both methods are increasingly being applied in environmental epidemiology to investigate the health effects of a mixture of chemical exposures [17,22]. Elastic net penalty regression is a method that calculates the penalty terms of the regression coefficients for each dietary variable, deleting irrelevant variables by shrinking their effect estimates exactly to zero [17]. In other words, a long list of inter-related food groups can be trimmed down, leaving us with a mixture of food groups related to the health outcomes. In addition, Quantile G-Computation analyzes mixtures data and generates the overall exposure effects of interest. Quantile G-Computation does not require pre-assigned positive or negative weights on a selected dietary variable [40], and has an advantage of time-to-event analysis with computational efficiency [10,22]. However, one limitation of Quantile G-Computation is that some exposures with small effects can be misclassified with regard to their effect direction [22]. For example, the Quantile G-Computation identified that mushroom and fungi have a positive weight on all-cause mortality among women, which does not agree with the existing evidence on the health benefits of mushroom and fungi [41]. However, the results from survival analysis did not find a significant positive association between mushroom and fungi intake and all-cause mortality among women (HR: 0.87, 95% CI = 0.67, 1.13). The Quantile G-Computation can be a supplementary approach to strengthen research findings from a conventional analytical approach (e.g., Cox proportional hazards models), which might have less predictive power when handling multicomponent exposures.

### 4.4. Strengths and Limitations

The strengths of our study include the long-term (>10 years) prospective cohort design with mortality outcomes being followed-up. With the use of sophisticated statistical methods, we have assessed the contribution of each food group on mortality risk, and the joint effect of food consumption. However, we should interpret the observations by taking note of several limitations. First, participants in the OS Study were well-educated compared with the general Hong Kong population, which might limit the generalizability of the results. Second, dietary data was self-reported, which might introduce recall bias especially for older adults. In addition, the changes in diet and lifestyle factors during follow-up, and the incidence of comorbidities were not determined. Lastly, the influence of residual confounding cannot be ruled out.

## 5. Conclusions

This study has quantified the relative importance of individual food groups in a mixture of exposures with the risk of all-cause and cause-specific mortalities. Whole grains, legumes, fruits, mushroom and fungi, and soy and soy products were the main contributors to the reduction of mortality risk among Chinese community-dwelling older adults. Moreover, the sex-specific association of sweets and desserts with cancer mortality may be worth further investigation. More prospective cohorts should adopt sophisticated statistical methods that can investigate the single and joint effects of dietary components on long-term health.

## Figures and Tables

**Table 1:** Baseline characteristics of participants in the Mr. OS and Ms. OS Study.

	Men (*n* = 1998)	Women (*n* = 1997)	*p* Value
	Mean (SD)/N (%)		
Age (Years)	72.4 ± 5.01	72.6 ± 5.36	0.618
Post-secondary Education	286 (14.3)	130 (6.5)	<0.001
Physical activity (PASE score)	97.3 ± 50.3	85.3 ± 33.1	<0.001
Smoking habit			<0.001
Former smoker	1037 (51.9)	153 (7.7)	
Current smoker	237 (11.9)	37 (1.9)	
Drink > 12 alcoholic drinks in the past year	471 (23.6)	51 (2.6)	<0.001
Dietary energy (kcal)	2100 ± 587	1580 ± 462	<0.001
Body mass index (kg/m^2^)	23.5 ± 3.13	23.9 ± 3.45	<0.001
History of diabetes	293 (14.7)	286 (14.3)	0.792
History of hypertension	836 (41.8)	869 (43.5)	0.300
History of stroke	108 (5.4)	65 (3.3)	0.001
History of heart attack	201 (10.1)	192 (9.6)	0.675
History of angina	205 (10.3)	147 (7.4)	0.001
History of congestive heart failure	73 (3.7)	78 (3.9)	0.738
History of cancer	87 (4.4)	90 (4.5)	0.875

χ^2^ test (categorical variables) and Mann–Whitney *U* test (continuous variables) for subgroup differences. *p* < 0.05. Abbreviations: PASE (Physical Activity Scale for the Elderly); SD: standard deviation; N, the sample size.

**Table 2:** Prospective association of quartile (Q) of food groups with an all-cause mortality risk selected by elastic net regression among participants in the Mr. OS and Ms. OS Study.

Foods (g/Day)	HR (95% CI)				*p*-Value for Trend
	Q1	Q2	Q3	Q4	
Dark green and leafy vegetables					
Overall	1.00	0.87 (0.75, 1.01)	0.84 (0.72, 0.97)	0.82 (0.70, 0.96)	0.049
Men	1.00	0.84 (0.70, 1.01)	0.83 (0.69, 1.01)	0.82 (0.67, 0.99)	0.256
Women	1.00	0.93 (0.73, 1.18)	0.82 (0.64, 1.05)	0.83 (0.65, 1.08)	0.144
Dim sum ^#^					
Overall	1.00	0.81 (0.69, 0.94)	0.88 (0.76, 1.03)	0.88 (0.75, 1.03)	0.414
Men	1.00	0.76 (0.62, 0.94)	0.78 (0.63, 0.96)	0.88 (0.73, 1.07)	0.161
Women	1.00	0.87 (0.69, 1.08)	1.05 (0.84, 1.32)	0.76 (0.56, 1.03)	0.081
Fruit					
Overall	1.00	0.93 (0.80, 1.08)	0.90 (0.77, 1.04)	0.79 (0.68, 0.93)	0.006
Men	1.00	0.95 (0.78, 1.15)	0.91 (0.75, 1.11)	0.79 (0.65, 0.96)	0.388
Women	1.00	0.89 (0.71, 1.13)	0.86 (0.68, 1.09)	0.84 (0.63, 1.10)	0.008
Legumes					
Overall	1.00	1.01 (0.87, 1.16)	0.79 (0.68, 0.92)	0.75 (0.63, 0.87)	0.052
Men	1.00	0.97 (0.80, 1.16)	0.77 (0.64, 0.94)	0.80 (0.65, 0.98)	0.023
Women	1.00	1.10 (0.88, 1.38)	0.84 (0.66, 1.07)	0.65 (0.50, 0.86)	0.626
Milk and milk products—high fat					
Overall	1.00	0.99 (0.84, 1.17)	0.93 (0.79, 1.09)	1.01 (0.86, 1.19)	0.107
Men	1.00	0.96 (0.76, 1.19)	0.89 (0.71, 1.11)	0.94 (0.75, 1.18)	0.852
Women	1.00	0.98 (0.76, 1.27)	0.98 (0.77, 1.24)	1.08 (0.85, 1.38)	0.129
Mushroom and fungi					
Overall	1.00	0.84 (0.72, 0.97)	0.75 (0.65, 0.87)	0.76 (0.65, 0.88)	0.023
Men	1.00	0.83 (0.69, 1.00)	0.72 (0.60, 0.87)	0.69 (0.57, 0.84)	0.553
Women	1.00	0.82 (0.65, 1.03)	0.78 (0.61, 0.99)	0.87 (0.67, 1.13)	0.023
Refined grains					
Overall	1.00	1.06 (0.90, 1.24)	1.05 (0.89, 1.24)	1.13 (0.95, 1.34)	0.088
Men	1.00	1.00 (0.80, 1.25)	1.02 (0.82, 1.28)	1.15 (0.92, 1.44)	0.393
Women	1.00	1.10 (0.88, 1.37)	1.07 (0.84, 1.36)	0.95 (0.70, 1.31)	0.012
Soy and soy products					
Overall	1.00	0.89 (0.77, 1.02)	0.79 (0.68, 0.92)	0.77 (0.66, 0.90)	0.143
Men	1.00	0.90 (0.74, 1.08)	0.76 (0.63, 0.93)	0.79 (0.65, 0.96)	0.073
Women	1.00	0.86 (0.69, 1.09)	0.87 (0.68, 1.10)	0.75 (0.57, 0.99)	0.590
Tea					
Overall	1.00	0.99 (0.85, 1.16)	1.03 (0.89, 1.21)	1.01 (0.87, 1.19)	0.760
Men	1.00	1.02 (0.82, 1.27)	1.16 (0.94, 1.44)	1.00 (0.81, 1.23)	0.554
Women	1.00	0.94 (0.75, 1.18)	0.88 (0.69, 1.11)	1.20 (0.92, 1.58)	0.965
Whole grains					
Overall	1.00	0.91 (0.79, 1.06)	0.85 (0.73, 0.98)	0.76 (0.65, 0.89)	0.008
Men	1.00	0.92 (0.77, 1.10)	0.82 (0.68, 1.00)	0.79 (0.64, 0.98)	0.059
Women	1.00	0.89 (0.68, 1.16)	0.87 (0.68, 1.11)	0.72 (0.56, 0.92)	0.058

Abbreviations: HR, hazard ratio; CI, confidence interval. *p* < 0.05. # Dim sum is a range of small Chinese dishes that are usually consumed in traditional Chinese restaurants. The Cox regression model was adjusted for sex, age, dietary energy, body mass index, physical activity, systolic blood pressure, and medical history (diabetes, hypertension, stroke, heart attack, angina, congestive heart failure or cancer), smoking habits, alcohol drinking, and education level.

**Table 3:** Prospective association of the quartile (Q) of food groups with cardiovascular mortality selected by elastic net regression among participants in the Mr. OS and Ms. OS Study.

Foods (g/Day)	HR (95% CI)				*p*-Value for Trend
	Q1	Q2	Q3	Q4	
Legumes					
Overall	1.00	0.98 (0.73, 1.32)	0.81 (0.59, 1.10)	0.64 (0.45, 0.91)	0.065
Men	1.00	0.76 (0.52, 1.13)	0.59 (0.39, 0.88)	0.62 (0.41, 0.94)	0.094
Women	1.00	1.44 (0.91, 2.30)	1.28 (0.78, 2.09)	0.62 (0.33, 1.15)	0.334
Starchy vegetables					
Overall	1.00	1.00 (0.74, 1.34)	0.71 (0.52, 0.99)	0.81 (0.59, 1.12)	0.085
Men	1.00	0.79 (0.54, 1.17)	0.69 (0.45, 1.05)	0.68 (0.45, 1.03)	0.227
Women	1.00	1.34 (0.84, 2.12)	0.72 (0.42, 1.24)	1.00 (0.60, 1.65)	0.170
Tomatoes					
Overall	1.00	1.08 (0.80, 1.45)	0.98 (0.72, 1.34)	0.74 (0.52, 1.04)	0.039
Men	1.00	1.10 (0.74, 1.65)	1.00 (0.67, 1.51)	0.80 (0.52, 1.23)	0.086
Women	1.00	1.02 (0.66, 1.60)	0.92 (0.56, 1.51)	0.59 (0.33, 1.07)	0.211

Abbreviations: HR, hazard ratio; CI, confidence interval. *p* < 0.05. The Cox regression model was adjusted for sex, age, dietary energy, body mass index, physical activity, systolic blood pressure, medical history (diabetes, hypertension, stroke, heart attack, angina, congestive heart failure or cancer), smoking habits, alcohol drinking, and education level.

**Table 4:** Prospective association of the quartile (Q) of food groups with cancer mortality selected by Elastic net regression among participants in the Mr. OS and Ms. OS Study.

Foods (g/Day)	HR (95% CI)				*p*-Value for Trend
	Q1	Q2	Q3	Q4	
Dim sum #					
Overall	1.00	0.95 (0.73, 1.24)	0.93 (0.71, 1.22)	1.02 (0.78, 1.34)	0.710
Men	1.00	0.86 (0.59, 1.24)	0.76 (0.53, 1.10)	1.00 (0.72, 1.38)	0.463
Women	1.00	1.06 (0.71, 1.57)	1.20 (0.81, 1.78)	0.85 (0.51, 1.43)	0.440
Fats and oils					
Overall	1.00	1.02 (0.77, 1.36)	1.15 (0.87, 1.53)	1.10 (0.81, 1.48)	0.974
Men	1.00	0.94 (0.62, 1.44)	1.02 (0.68, 1.52)	1.00 (0.66, 1.49)	0.774
Women	1.00	1.02 (0.69, 1.51)	1.31 (0.86, 1.98)	1.14 (0.68, 1.88)	0.596
Fruit					
Overall	1.00	0.94 (0.73, 1.20)	0.79 (0.61, 1.02)	0.68 (0.51, 0.89)	0.002
Men	1.00	0.92 (0.67, 1.26)	0.84 (0.61, 1.16)	0.62 (0.44, 0.88)	0.001
Women	1.00	0.98 (0.66, 1.46)	0.73 (0.47, 1.13)	0.84 (0.52, 1.35)	0.620
Nuts					
Overall	1.00	0.84 (0.65, 1.08)	0.87 (0.68, 1.12)	0.72 (0.55, 0.94)	0.550
Men	1.00	0.87 (0.62, 1.21)	0.93 (0.67, 1.28)	0.67 (0.48, 0.94)	0.776
Women	1.00	0.81 (0.54, 1.20)	0.79 (0.52, 1.19)	0.84 (0.54, 1.33)	0.193
Other vegetables					
Overall	1.00	0.89 (0.69, 1.15)	0.96 (0.75, 1.24)	0.84 (0.64, 1.10)	0.100
Men	1.00	0.81 (0.59, 1.11)	0.76 (0.55, 1.05)	0.78 (0.56, 1.09)	0.051
Women	1.00	1.12 (0.72, 1.75)	1.48 (0.96, 2.26)	1.03 (0.64, 1.67)	0.958
Refined grains					
Overall	1.00	1.11 (0.85, 1.45)	0.86 (0.64, 1.15)	1.19 (0.89, 1.60)	0.148
Men	1.00	1.00 (0.69, 1.46)	0.74 (0.50, 1.11)	1.23 (0.85, 1.78)	0.016
Women	1.00	1.22 (0.83, 1.79)	1.03 (0.67, 1.59)	0.89 (0.51, 1.56)	0.378
Soup					
Overall	1.00	0.77 (0.59, 1.00)	0.75 (0.58, 0.98)	0.89 (0.69, 1.15)	0.258
Men	1.00	0.71 (0.50, 1.01)	0.73 (0.52, 1.03)	0.88 (0.64, 1.21)	0.062
Women	1.00	0.87 (0.58, 1.28)	0.80 (0.52, 1.23)	0.91 (0.58, 1.43)	0.724
Soy and soy products					
Overall	1.00	0.98 (0.76, 1.25)	0.89 (0.69, 1.15)	0.72 (0.54, 0.95)	0.051
Men	1.00	0.95 (0.69, 1.31)	0.87 (0.62, 1.20)	0.69 (0.49, 0.98)	0.196
Women	1.00	1.04 (0.70, 1.55)	0.96 (0.63, 1.46)	0.79 (0.49, 1.28)	0.130
Sweets and desserts					
Overall	1.00	0.99 (0.75, 1.30)	0.77 (0.58, 1.03)	0.99 (0.75, 1.32)	0.478
Men	1.00	1.23 (0.83, 1.84)	1.03 (0.68, 1.56)	1.37 (0.91, 2.05)	0.325
Women	1.00	0.89 (0.60, 1.33)	0.62 (0.40, 0.96)	0.68 (0.42, 1.09)	0.629
Tea					
Overall	1.00	1.26 (0.95, 1.68)	1.23 (0.92, 1.63)	1.44 (1.09, 1.90)	0.077
Men	1.00	1.02 (0.69, 1.51)	1.15 (0.79, 1.67)	1.11 (0.78, 1.59)	0.679
Women	1.00	1.44 (0.95, 2.18)	1.18 (0.76, 1.85)	2.16 (1.38, 3.37)	0.013
Water					
Overall	1.00	0.94 (0.73, 1.21)	0.77 (0.59, 1.00)	0.89 (0.69, 1.14)	0.176
Men	1.00	0.92 (0.66, 1.27)	0.89 (0.65, 1.23)	0.98 (0.72, 1.34)	0.725
Women	1.00	0.95 (0.62, 1.44)	0.62 (0.39, 0.98)	0.75 (0.48, 1.17)	0.102
Whole grains					
Overall	1.00	0.99 (0.77, 1.27)	0.83 (0.64, 1.08)	0.79 (0.60, 1.05)	0.078
Men	1.00	0.99 (0.73, 1.34)	0.86 (0.61, 1.20)	0.78 (0.53, 1.12)	0.026
Women	1.00	0.98 (0.63, 1.52)	0.77 (0.50, 1.19)	0.79 (0.52, 1.22)	0.980

Abbreviations: HR, hazard ratio; CI, confidence interval. *p* < 0.05. # Dim sum is a range of small Chinese dishes that are usually consumed in traditional Chinese restaurants. The Cox regression model was adjusted for sex, age, dietary energy, body mass index, physical activity, systolic blood pressure, medical history (diabetes, hypertension, stroke, heart attack, angina, congestive heart failure or cancer), smoking habits, alcohol drinking, and education level.

## Data Availability

All relevant data are within the manuscript.

## Supplementary Materials

The following supporting information can be downloaded at: https://www.mdpi.com/article/10.3390/nu14193915/s1, Table S1: Study checklist using STROBE-nut; Table S2: Dietary consumption of Participants in the Mr. OS and Ms. OS Study by the quartiles (Q) of intake; Table S3: Median dietary consumption of each food group for overall/men/women; Table S4: Coefficients of Elastic Net Regression models on the associations between food groups and cause-specific mortality among Participants in Mr. OS and Ms. OS Study; Table S5: The number of all-cause mortality by quartile (Q) of food groups selected by Elastic net regression among participants in the Mr. OS and Ms. OS Study; Table S6: The number of cardiovascular mortality by the quartile (Q) of food groups selected by Elastic net regression among participants in the Mr. OS and Ms. OS Study; Table S7: The number of cancer mortality by the quartile (Q) of food groups selected by Elastic net regression among participants in the Mr. OS and Ms. OS Study; Figure S1: The chart of analytic study design; Figure S2: Elastic net analysis on the association of food groups and all-cause mortality; Figure S3: Elastic net analysis on the association of food groups and CVD mortality; Figure S4: Elastic net analysis on the association of food groups and cancer mortality.
