# Supplementary material for: Joint Associations of Food Groups with All-Cause and Cause-Specific Mortality in the Mr. OS and Ms. OS Study: A Prospective Cohort
Source: Nutrients. 2022 Sep 21;14(19):3915. doi: 10.3390/nu14193915 (PMC9573629; doi:10.3390/nu14193915)
Supplement: Supplementary file 1 [file nutrients-14-03915-s001.zip › nutrients-1891201-supplementary.pdf]

Supplementary Materials

Joint Associations of Food Groups with All-Cause and Cause-Specific Mortality in the Mr. OS and Ms. OS Study: A Prospective Cohort

Jingli Yang [1,2,†], Aimin Yang [3,†], Suey Yeung [3], Jean Woo [3,*] and Kenneth Lo [4,5,*]

[1] College of Earth and Environmental Sciences, Lanzhou University, Lanzhou 730000, China
[2] School of Public Health and Social Work, Queensland University of Technology, Brisbane 4059, Australia
[3] Department of Medicine and Therapeutics, The Chinese University of Hong Kong, Shatin, New Territories, Hong Kong SAR, China
[4] Department of Applied Biology and Chemical Technology, The Hong Kong Polytechnic University, 11 Yuk Choi Road, Hung Hom, Kowloon, Hong Kong SAR, China
[5] Research Institute for Smart Ageing, The Hong Kong Polytechnic University, Hong Kong SAR, China
* Correspondence: jeanwoowong@cuhk.edu.hk (J.W.); khklo@polyu.edu.hk (K.L.)
† These authors contributed equally to this work.

Table of Contents

Table S2. Dietary consumption of Participants in the Mr OS and Ms OS Study by the quartiles (Q) of intake 11

Table S3. Median dietary consumption of each food group for overall/men/women 15

Table S4. Coefficients of Elastic Net Regression models on the associations between food groups and cause-specific mortality among Participants in Mr OS and Ms OS Study 17

Table S5. Prospective association of quartile (Q) of food groups with all-cause mortality risk se-lected by Elastic net regression among Participants in the Mr OS and Ms OS Study 18

Table S6. Prospective Association of the quartile (Q) of food groups with Cardiovascular Mortality selected by Elastic net regression Among Participants in the Mr OS and Ms OS Study 19

Table S7. Prospective Association of the quartile (Q) of food groups with Cancer Mortality selected by Elastic Net Regression Among Participants in the Mr OS and Ms OS Study 20

Figure S1. The chart of analytic study design 22

Figure S2. Elastic net analysis on the association of food groups and all-cause mortality. 23

Figure S3. Elastic net analysis on the association of food groups and CVD mortality. 24

Figure S4. Elastic net analysis on the association of food groups and cancer mortality 25

Table S1. Study checklist using STROBE-nut

Item	Item nr	STROBE recommendations	Extension for Nutritional Epidemiology studies (STROBE-nut)	Reported on page #
**Title and abstract**	1	(a) Indicate the study's design with a commonly used term in the title or the abstract.<br>(b) Provide in the abstract an informative and balanced summary of what was done and what was found.	**nut-1** State the dietary/nutritional assessment method(s) used in the title, abstract, or keywords.	**1-3**
**Introduction**				
Background rationale	2	Explain the scientific background and rationale for the investigation being reported.		3-4
Objectives	3	State specific objectives, including any pre-specified hypotheses.		3-4
**Methods**				
Study design	4	Present key elements of study design early in the paper.		4-5
Settings	5	Describe the setting, locations, and relevant dates, including periods of recruitment, exposure, follow-up, and data collection.	**nut-5** Describe any characteristics of the study settings that might affect the dietary intake or nutritional status of the participants, if applicable.	**4-5**
Participants	6	a) Cohort study—Give the eligibility criteria, and the sources and methods of selection of participants. Describe methods of follow-up.	**nut-6** Report particular dietary, physiological or nutritional characteristics that were considered	**4-5**

Item	Item nr	STROBE recommendations	Extension for Nutritional Epidemiology studies (STROBE-nut)	Reported on page #
		Case-control study—Give the eligibility criteria, and the sources and methods of case ascertainment and control selection. Give the rationale for the choice of cases and controls. Cross-sectional study—Give the eligibility criteria, and the sources and methods of selection of participants. (b) Cohort study—For matched studies, give matching criteria and number of exposed and unexposed. Case-control study—For matched studies, give matching criteria and the number of controls per case.	when selecting the target population.	
Variables	7	Clearly define all outcomes, exposures, predictors, potential confounders, and effect modifiers. Give diagnostic criteria, if applicable.	**nut-7.1** Clearly define foods, food groups, nutrients, or other food components. **nut-7.2** When using dietary patterns or indices, describe the methods to obtain them and their nutritional properties.	**5-6**
Data sources - measurements	8	For each variable of interest, give sources of data and details of methods of assessment	**nut-8.1** Describe the dietary assessment method(s), e.g., portion size	**5-6**

Item	Item nr	STROBE recommendations	Extension for Nutritional Epidemiology studies (STROBE-nut)	Reported on page #
		(measurement).Describe comparability of assessment methods if there is more than one group.	estimation, number of days and items recorded, how it was developed and administered, and how quality was assured. Report if and how supplement intake was assessed. **nut-8.2** Describe and justify food composition data used. Explain the procedure to match food composition with consumption data. Describe the use of conversion factors, if applicable. **nut-8.3** Describe the nutrient requirements, recommendations, or dietary guidelines and the evaluation approach used to compare intake with the dietary reference values, if applicable. **nut-8.4** When using nutritional biomarkers, additionally use the STROBE Extension for Molecular Epidemiology (STROBE-ME). Report the type of	

Item	Item nr	STROBE recommendations	Extension for Nutritional Epidemiology studies (STROBE-nut)	Reported on page #
			biomarkers used and their usefulness as dietary exposure markers.<br>**nut-8.5** Describe the assessment of nondietary data (e.g., nutritional status and influencing factors) and timing of the assessment of these variables in relation to dietary assessment.<br>**nut-8.6** Report on the validity of the dietary or nutritional assessment methods and any internal or external validation used in the study, if applicable.	
Bias	9	Describe any efforts to address potential sources of bias.	**nut-9** Report how bias in dietary or nutritional assessment was addressed, e.g., misreporting, changes in habits as a result of being measured, or data imputation from other sources	**5-6**
Study Size	10	Explain how the study size was arrived at.		5
Quantitative variables	11	Explain how quantitative variables were handled in the analyses. If applicable,	**nut-11** Explain categorization of dietary/nutritional	**5-6**

Item	Item nr	STROBE recommendations	Extension for Nutritional Epidemiology studies (STROBE-nut)	Reported on page #
		describe which groupings were chosen and why.	data (e.g., use of N-tiles and handling of nonconsumers) and the choice of reference category, if applicable.	
Statistical Methods	12	(a) Describe all statistical methods, including those used to control for confounding<br>(b) Describe any methods used to examine subgroups and interactions.<br>(c) Explain how missing data were addressed.<br>(d) Cohort study—If applicable, explain how loss to follow-up was addressed.<br>Case-control study—If applicable, explain how matching of cases and controls was addressed.<br>Cross-sectional study—If applicable, describe analytical methods taking account of sampling strategy.<br>(e) Describe any sensitivity analyses.	**nut-12.1** Describe any statistical method used to combine dietary or nutritional data, if applicable.<br>**nut-12.2** Describe and justify the method for energy adjustments, intake modeling, and use of weighting factors, if applicable.<br>**nut-12.3** Report any adjustments for measurement error, i.e,. from a validity or calibration study.	**6-8**
**Results**				
Participants	13	(a) Report the numbers of individuals at each stage of the study—e.g., numbers potentially eligible, examined for eligibility, confirmed	**nut-13** Report the number of individuals excluded based on missing, incomplete or implausible	**8-9**

Item	Item nr	STROBE recommendations	Extension for Nutritional Epidemiology studies (STROBE-nut)	Reported on page #
		eligible, included in the study, completing follow-up, and analyzed.<br>(b) Give reasons for non-participation at each stage.<br>(c) Consider use of a flow diagram.	dietary/nutritional data.	
Descriptive data	14	(a) Give characteristics of study participants (e.g., demographic, clinical, social) and information on exposures and potential confounders<br>(b) Indicate the number of participants with missing data for each variable of interest<br>(c) Cohort study—Summarize follow-up time (e.g., average and total amount)	**nut-14** Give the distribution of participant characteristics across the exposure variables if applicable. Specify if food consumption of total population or consumers only were used to obtain results.	**8-9**
Outcome data	15	Cohort study—Report numbers of outcome events or summary measures over time.<br>Case-control study—Report numbers in each exposure category, or summary measures of exposure.<br>Cross-sectional study—Report numbers of outcome events or summary measures.		9-10
Main results	16	(a) Give unadjusted estimates and, if applicable, confounder-	**nut-16** Specify if nutrient intakes are reported with or	**9-10**

Item	Item nr	STROBE recommendations	Extension for Nutritional Epidemiology studies (STROBE-nut)	Reported on page #
		adjusted estimates and their precision (e.g., 95% confidence interval). Make clear which confounders were adjusted for and why they were included. (b) Report category boundaries when continuous variables were categorized. (c) If relevant, consider translating estimates of relative risk into absolute risk for a meaningful time period.	without inclusion of dietary supplement intake, if applicable.	
Other analyses	17	Report other analyses done—e.g., analyses of subgroups and interactions and sensitivity analyses.	**nut-17** Report any sensitivity analysis (e.g., exclusion of misreporters or outliers) and data imputation, if applicable.	**10-12**
**Discussion**				
Key results	18	Summarize key results with reference to study objectives.		12
Limitation	19	Discuss limitations of the study, taking into account sources of potential bias or imprecision. Discuss both direction and magnitude of any potential bias.	**nut-19** Describe the main limitations of the data sources and assessment methods used and implications for the interpretation of the findings.	**16-17**
Interpretation	20	Give a cautious overall interpretation of results considering objectives,	**nut-20** Report the nutritional relevance of the findings, given	**12-16**

Item	Item nr	STROBE recommendations	Extension for Nutritional Epidemiology studies (STROBE-nut)	Reported on page #
		limitations, multiplicity of analyses, results from similar studies, and other relevant evidence.	the complexity of diet or nutrition as an exposure.	
Generalizability	21	Discuss the generalizability (external validity) of the study results.		16-17
**Other information**				
Funding	22	Give the source of funding and the role of the funders for the present study and, if applicable, for the original study on which the present article is based.		**18**
Ethics			**nut-22.1** Describe the procedure for consent and study approval from ethics committee(s).	**5,17**
Supplementary material			**nut-22.2** Provide data collection tools and data as online material or explain how they can be accessed.	**Additional File 1**

Table S2. Dietary consumption of Participants in the Mr OS and Ms OS Study by the quartiles (Q) of intake

Foods (g/day)	Q1	Q2	Q3	Q4
**Included food groups**				
**Cakes, cookies, pies and biscuits**				
Overall	< 6.59	6.59 – 14.29	14.29 – 28.30	> 28.30
Men	< 7.97	7.97 – 16.95	16.95 – 32.14	> 32.14
Women	< 5.38	5.38 – 11.92	11.92 – 24.64	> 24.64
**Cruciferous vegetables**				
Overall	< 6.59	6.59 – 15.38	15.38 – 30.22	> 30.22
Men	< 6.83	6.83 – 16.32	16.32 – 30.22	> 30.22
Women	< 6.59	6.59 – 15.38	15.38 – 30.22	> 30.22
**Dark green and leafy vegetables**				
Overall	< 41.21	41.21 – 67.03	67.03 – 101.92	> 101.92
Men	< 37.40	37.40 – 64.29	64.29 – 100.55	> 100.55
Women	< 44.51	44.51 – 68.13	68.13 – 103.57	> 130.57
**Dim sum**[#]				
Overall	< 9.73	9.73 – 26.26	26.26 – 57.24	> 57.24
Men	< 14.85	14.85 – 37.03	37.03 – 78.24	> 78.24
Women	< 7.09	7.09 – 18.87	18.87 – 39.78	> 39.78
**Eggs**				
Overall	< 3.85	3.85 – 7.97	7.97 – 15.11	> 15.11
Men	< 3.85	3.85 – 8.79	8.79 – 15.93	> 15.93
Women	< 3.85	3.85 – 7.69	7.69 – 14.56	> 14.56
**Fats and oils**				
Overall	< 16.62	16.62 – 21.06	21.06 – 28.08	> 28.08
Men	< 18.72	18.72 – 23.89	23.89 – 29.41	> 29.41
Women	< 14.04	14.04 – 18.72	18.72 – 25.74	> 25.74
**Fish and seafood**				
Overall	< 35.71	35.71 – 62.50	62.50 – 100.63	> 100.63
Men	< 39.19	39.19 – 69.73	69.73 – 116.52	> 116.52
Women	< 32.09	32.09 – 57.23	57.23 – 88.74	> 88.74
**Fruit**				
Overall	< 154.23	154.23 – 228.63	228.63 – 334.98	> 334.98
Men	< 160.30	160.30 – 238.06	238.06 – 352.52	> 352.52
Women	< 150.21	150.21 – 218.24	218.24 – 320.95	> 320.95
**Legumes**				
Overall	< 4.26	4.26 – 9.34	9.34 – 19.02	> 19.02
Men	< 4.53	4.53 – 9.57	9.57 – 18.37	> 18.37
Women	< 4.12	4.12 – 9.23	9.23 – 19.23	> 19.23
**Milk and milk products - high fat**				
Overall	0.00	0 – 2.70	2.70 – 14.41	> 14.41
Men	0.00	0 – 4.41	4.41 – 19.20	> 19.20
Women	0.00	0 – 2.21	2.21 – 10.31	> 10.31

**Mushroom and fungi**				
Overall	< 0.40	0.40 – 1.64	1.64 – 4.48	> 4.48
Men	< 0.48	0.48 – 1.97	1.97 – 5.16	> 5.16
Women	< 0.34	0.34 – 1.28	1.28 – 3.95	> 3.95
**Nuts**				
Overall	< 0.21	0.21 – 1.63	1.63 – 4.23	> 4.23
Men	< 0.41	0.41 – 1.98	1.98 – 5.12	> 5.12
Women	< 0.00	0.00 – 1.15	1.15 – 3.54	> 3.54
**Other vegetables**				
Overall	< 46.36	46.36 – 73.59	73.59 – 111.10	> 111.10
Men	< 46.43	46.43 – 73.90	73.90 – 114.29	> 114.29
Women	< 46.34	46.34 – 73.31	73.31 – 108.52	> 108.52
**Poultry**				
Overall	< 7.69	7.69 – 15.66	15.66 – 30.22	> 30.22
Men	< 9.78	9.78 – 18.68	18.68 – 35.97	> 35.97
Women	< 6.20	6.20 – 12.98	12.98 – 24.42	> 24.42
**Red and processed meat**				
Overall	< 18.61	18.61 – 34.13	34.13 – 58.29	> 58.29
Men	< 25.21	25.21 – 44.53	44.53 – 72.38	> 72.38
Women	< 14.29	14.29 – 26.72	26.72 – 44.34	> 44.34
**Refined grains**				
Overall	< 396.48	396.48 – 488.57	488.57 – 633.85	> 633.85
Men	< 434.09	434.09 – 538.46	538.46 – 715.38	> 715.38
Women	< 356.86	356.86 – 452.58	452.58 – 548.46	> 548.46
**Soup**				
Overall	< 61.54	61.54 – 128.57	128.57 – 232.97	> 232.97
Men	< 83.52	83.52 – 164.29	164.29 – 278.02	> 278.02
Women	< 57.14	57.14 – 114.29	114.29 – 200.55	> 200.55
**Soy and soy products**				
Overall	< 14.26	14.26 – 33.52	33.52 – 66.21	> 66.21
Men	< 15.66	15.66 – 36.68	36.68 – 73.22	> 73.22
Women	< 12.98	12.98 – 31.21	31.21 – 59.89	> 59.89
**Starchy vegetables**				
Overall	< 1.65	1.65 – 4.67	4.67 – 10.44	> 10.44
Men	< 1.65	1.65 – 4.67	4.67 – 10.16	> 10.16
Women	< 1.65	1.65 – 4.84	4.84 – 10.99	> 10.99
**Sweets and desserts**				
Overall	0.00	0.00 – 6.59	6.59 – 15.38	> 15.38
Men	0.00	0.00 – 6.59	6.59 – 16.81	> 16.81
Women	0.00	0.00 – 3.30	3.30 – 13.19	> 13.19
**Tea**				
Overall	< 65.93	65.93 – 376.03	376.03 – 752.06	> 752.06
Men	< 178.57	178.57 – 501.37	501.37 – 1002.7	> 1002.74
Women	< 16.48	16.48 – 250.69	250.69 – 505.49	> 505.49
**Tomatoes**				
Overall	< 3.30	3.30 – 14.29	14.29 – 23.08	> 23.08
Men	< 3.85	3.85 – 14.29	14.29 – 28.57	> 28.57

Women	< 3.30	3.030 – 11.54	11.54 – 21.43	> 21.43
**Water**				
Overall	< 626.72	626.72 – 1002.7	1002.7 – 1378.8	> 1378.8
Men	< 501.37	501.37 – 1002.7	1002.7 – 1281.3	> 1281.3
Women	< 752.06	752.06 –1002.7	1002.7 – 1504.1	> 1504.1
**Whole grain**				
Overall	0.00	0.00 – 16.71	16.71 – 80.22	> 80.22
Men	0.00	0.00 – 6.59	6.59 – 57.14	> 57.14
Women	0.00	0.00 – 29.23	29.23 – 88.57	> 88.57
**Excluded food groups**				
**Beer and wine**				
Overall	0.00	0.00 – 0.00	0.00 – 0.00	> 0.00
Men	0.00	0.00 – 0.00	0.00 – 4.12	> 4.12
Women	0.00	0.00 – 0.00	0.00 – 0.00	> 0.00
**Beverages**				
Overall	0.00	0.00 – 0.00	0.00 – 8.24	> 8.24
Men	0.00	0.00 – 0.38	0.38 – 12.11	> 12.11
Women	0.00	0.00 – 0.00	0.00 – 4.12	> 4.12
**Coffee**				
Overall	0.00	0.00 – 0.00	0.00 – 0.00	> 0.00
Men	0.00	0.00 – 0.00	0.00 – 8.24	> 8.24
Women	0.00	0.00 – 0.00	0.00 – 0.00	> 0.00
**Condiments**				
Overall	0.00	0.00 – 0.00	0.00 – 2.86	> 2.86
Men	0.00	0.00 – 0.00	0.00 – 4.00	> 4.00
Women	0.00	0.00 – 0.00	0.00 – 1.03	> 1.03
**Fast foods**				
Overall	0.00	0.00 – 0.00	0 – 4.32	> 4.32
Men	0.00	0.00 – 0.00	0 – 4.68	> 4.68
Women	0.00	0.00 – 0.00	0 – 3.36	> 3.36
**French fries and potato chips**				
Overall	0.00	0.00 – 0.00	0.00 – 0.00	> 0.00
Men	0.00	0.00 – 0.00	0.00 – 0.00	> 0.00
Women	0.00	0.00 – 0.00	0.00 – 0.00	> 0.00
**Organ meats**				
Overall	0.00	0.00 – 0.00	0.00 – 0.41	> 0.41
Men	0.00	0.00 – 0.00	0.00 – 1.65	> 1.65
Women	0.00	0.00 – 0.00	0.00 – 0.00	> 0.00
**Preserved vegetables**				
Overall	0.00	0.00 – 0.00	0.00 – 0.71	> 0.71
Men	0.00	0.00 – 0.00	0.00 – 0.82	> 0.82
Women	0.00	0.00 – 0.00	0.00 – 0.55	> 0.55
**Milk and milk products - low fat**				
Overall	0.00	0.00 – 0.00	0.00 – 15.73	> 15.73
Men	0.00	0.00 – 0.00	0.00 – 12.00	> 12.00

Women	0.00	0.00 – 3.00	3.00 – 18.80	> 18.80
**Milk and milk products - yogurt**				
Overall	0.00	0.00 – 0.00	0.00 – 0.00	> 0.00
Men	0.00	0.00 – 0.00	0.00 – 0.00	> 0.00
Women	0.00	0.00 – 0.00	0.00 – 0.00	> 0.00

Abbreviations: Q (Quartile).

Dim sum is a range of small Chinese dishes that are usually consumed in traditional Chinese restaurants.

Table S3. Median dietary consumption of each food group for overall/men/women

**Foods (g/day)**	**Overall**, N = 3,995[1]	**Men**, N = 1,998[1]	**Women**, N = 1,997[1]	**p-value**[2]
**Cakes, cookies, pies and biscuits**	14 (7, 28)	17 (8, 32)	12 (5, 25)	<0.001
**Cruciferous vegetables**	15 (7, 30)	16 (7, 30)	15 (7, 30)	0.4
**Dark green and leafy vegetables**	67 (41, 102)	64 (37, 101)	68 (45, 104)	0.003
**Dim sum**	26 (10, 57)	37 (15, 78)	19 (7, 40)	<0.001
**Eggs**	8 (4, 15)	9 (4, 16)	8 (4, 15)	<0.001
**Fats and oils**	21 (17, 28)	24 (19, 29)	19 (14, 26)	<0.001
**Fish and seafood**	62 (36, 101)	70 (39, 117)	57 (32, 89)	<0.001
**Fruit**	229 (154, 335)	238 (160, 353)	218 (150, 321)	<0.001
**Legumes**	9 (4, 19)	10 (5, 18)	9 (4, 19)	0.3
**Milk and milk products - high fat**	3 (0, 14)	4 (0, 19)	2 (0, 10)	<0.001
**Mushroom and fungi**	1.6 (0.4, 4.5)	2.0 (0.5, 5.2)	1.3 (0.3, 3.9)	<0.001
**Nuts**	1.6 (0.2, 4.2)	2.0 (0.4, 5.2)	1.2 (0.0, 3.5)	<0.001
**Other vegetables**	74 (46, 111)	74 (46, 114)	73 (46, 109)	0.4
**Poultry**	16 (8, 30)	19 (10, 36)	13 (6, 24)	<0.001
**Red and processed meat**	34 (19, 58)	45 (25, 72)	27 (14, 44)	<0.001
**Refined grains**	489 (396, 634)	538 (434, 715)	453 (357, 548)	<0.001
**Soup**	129 (62, 233)	164 (84, 278)	114 (57, 201)	<0.001
**Soy and soy products**	34 (14, 66)	37 (16, 73)	31 (13, 60)	<0.001
**Starchy vegetables**	5 (2, 10)	5 (2, 10)	5 (2, 11)	0.3
**Sweets and desserts**	7 (0, 15)	7 (0, 17)	3 (0, 13)	<0.001
**Tea**	376 (66, 752)	501 (179, 1,003)	251 (16, 505)	<0.001

**Foods (g/day)**	**Overall**, N = 3,995[1]	**Men**, N = 1,998[1]	**Women**, N = 1,997[1]	**p-value**[2]
**Tomatoes**	14 (3, 23)	14 (4, 29)	12 (3, 21)	<0.001
**Water**	1,003 (627, 1,379)	1,003 (501, 1,281)	1,003 (752, 1,504)	<0.001
**Whole grain**	17 (0, 80)	7 (0, 57)	29 (4, 89)	<0.001

[1] Median (IQR)

[2] Wilcoxon rank sum test.

Table S4. Coefficients of Elastic Net Regression models on the associations between food groups and cause-specific mortality among Participants in Mr OS and Ms OS Study

	Mortality outcomes		
**Food groups**	All-cause	CVD	Cancer
Cakes, cookies, pies and biscuits	0.0000	0.0000	0.0000
Cruciferous vegetables	0.0000	0.0000	0.0000
Dark green and leafy vegetables	**-0.0008**[&]	0.0000	0.0000
Dim sum #	**0.0014**[&]	0.0000	**0.0005**[&]
Eggs	0.0000	0.0000	0.0000
Fats and oils	0.0000	0.0000	**0.0014**[&]
Fish and seafood	0.0000	0.0000	0.0000
Fruit	**-0.0003**[&]	0.0000	**-0.0005**[&]
Legumes	**-0.0015**[&]	**< -0.0001**[&]	0.0000
Milk and milk products - high fat	**0.0009**[&]	0.0000	0.0000
Mushroom and fungi	**-0.0033**[&]	0.0000	0.0000
Nuts	0.0000	0.0000	**0.0038**[&]
Other vegetables	0.0000	0.0000	**-0.0004**[&]
Poultry	0.0000	0.0000	0.0000
Red and processed meat	0.0000	0.0000	0.0000
Refined grains	**0.0002**[&]	0.0000	**0.0002**[&]
Soup	0.0000	0.0000	**0.0002**[&]
Soy and soy products	**-0.0002**[&]	0.0000	**-0.0005**[&]
Starchy vegetables	0.0000	**< -0.0001**[&]	0.0000
Sweets and desserts	0.0000	0.0000	**0.0006**[&]
Tea	**0.0002**[&]	0.0000	**0.0002**[&]
Tomatoes	0.0000	**< -0.0001**[&]	0.0000
Water	0.0000	0.0000	**-0.0001**[&]
Whole grain	**-0.0010**[&]	0.0000	**-0.0009**[&]

Dim sum is a range of small Chinese dishes that are usually consumed in traditional Chinese restaurants.

& Selected food groups with non-zero elastic net regression coefficients.

Table S5. The number of all-cause mortality by quartile (Q) of food groups selected by Elastic net regression among participants in the Mr OS and Ms OS Study

Foods, n/N	Q1	Q2	Q3	Q4
Dark green and leafy vegetables				
Overall	397/999	346/999	318/999	309/998
Men	266/555	201/476	196/488	189/479
Women	131/444	145/523	122/511	120/519
Dim sum				
Overall	360/999	306/999	328/999	376/998
Men	168/339	173/439	194/508	317/712
Women	192/660	133/560	134/491	59/286
Fruit				
Overall	384/999	341/999	336/999	309/998
Men	225/467	206/465	204/499	217/567
Women	159/532	135/534	132/500	92/431
Legumes				
Overall	389/999	373/999	326/999	282/998
Men	231/477	224/508	210/531	187/482
Women	158/522	149/491	116/468	95/516
Milk and milk products - high fat				
Overall	342/999	354/999	321/999	353/998
Men	158/324	249/583	204/500	241/591
Women	184/675	105/416	117/499	112/407
Mushroom and fungi				
Overall	424/999	340/999	306/999	300/998
Men	245/443	210/479	199/513	198/563
Women	179/556	130/520	107/486	102/435
Refined grains				
Overall	314/999	338/999	349/999	369/998
Men	149/346	180/426	219/510	304/716
Women	165/653	158/573	130/489	65/282
Soy and soy products				
Overall	402/999	352/999	308/999	308/998
Men	230/451	217/491	185/491	220/565
Women	172/548	135/508	123/508	88/433
Tea				
Overall	329/999	315/999	350/999	376/998
Men	140/323	181/449	234/512	297/714
Women	189/676	134/550	116/487	79/284
Whole grain				
Overall	407/999	353/999	322/999	288/998
Men	281/595	251/606	172/424	148/373
Women	126/404	102/393	150/575	140/625

Table S6. The number of cardiovascular mortality by the quartile (Q) of food groups selected by Elastic net regression among participants in the Mr OS and Ms OS Study

Foods, n/N	Q1	Q2	Q3	Q4
Legumes				
Overall	93/999	87/999	77/999	57/998
Men	60/477	46/508	42/531	40/482
Women	33/522	41/491	35/468	17/516
Starchy vegetables				
Overall	95/999	85/999	61/999	73/998
Men	59/484	47/521	39/506	43/487
Women	36/515	38/478	22/493	30/511
Tomatoes				
Overall	88/999	88/999	79/999	59/998
Men	47/452	49/478	50/545	42/523
Women	41/547	39/521	29/454	17/475

Table S7. The number of cancer mortality by the quartile (Q) of food groups selected by Elastic net regression among participants in the Mr OS and Ms OS Study

Foods, n/N	Q1	Q2	Q3	Q4
Dim sum				
Overall	108/999	108/999	111/999	142/998
Men	54/339	61/439	63/508	121/712
Women	54/660	47/560	48/491	21/286
Fats and oils				
Overall	95/999	107/999	134/999	133/998
Men	33/225	64/450	94/611	108/712
Women	62/774	43/549	40/388	25/286
Fruit				
Overall	134/999	127/999	110/999	98/998
Men	83/467	77/465	73/499	66/567
Women	51/532	50/534	37/500	32/431
Nuts				
Overall	130/999	113/999	120/999	106/998
Men	74/419	69/462	82/514	74/603
Women	56/580	44/537	38/485	32/395
Other vegetables				
Overall	129/999	114/999	121/999	105/998
Men	90/499	72/493	67/479	70/527
Women	39/500	42/506	54/520	35/471
Refined grains				
Overall	105/999	122/999	99/999	143/998
Men	52/346	66/426	58/510	123/716
Women	53/653	56/573	41/489	20/282
Soup				
Overall	122/999	106/999	106/999	135/998
Men	65/386	59/451	71/517	104/644
Women	57/613	47/548	35/482	31/354
Soy and soy products				
Overall	128/999	129/999	116/999	96/998
Men	78/451	81/491	73/491	67/565
Women	50/548	48/508	43/508	29/433
Sweets and desserts				
Overall	111/999	133/999	98/999	127/998
Men	41/316	91/559	68/533	99/590
Women	70/683	42/440	30/466	28/408
Tea				
Overall	86/999	110/999	115/999	158/998
Men	44/323	59/449	78/512	118/714
Women	42/676	51/550	37/487	40/284
Water				
Overall	149/999	113/999	97/999	110/998
Men	111/648	58/421	62/466	68/463
Women	38/351	55/578	35/533	42/535

Whole grain				
Overall	135/999	139/999	102/999	93/998
Men	94/595	101/606	58/424	46/373
Women	41/404	38/393	44/575	47/625

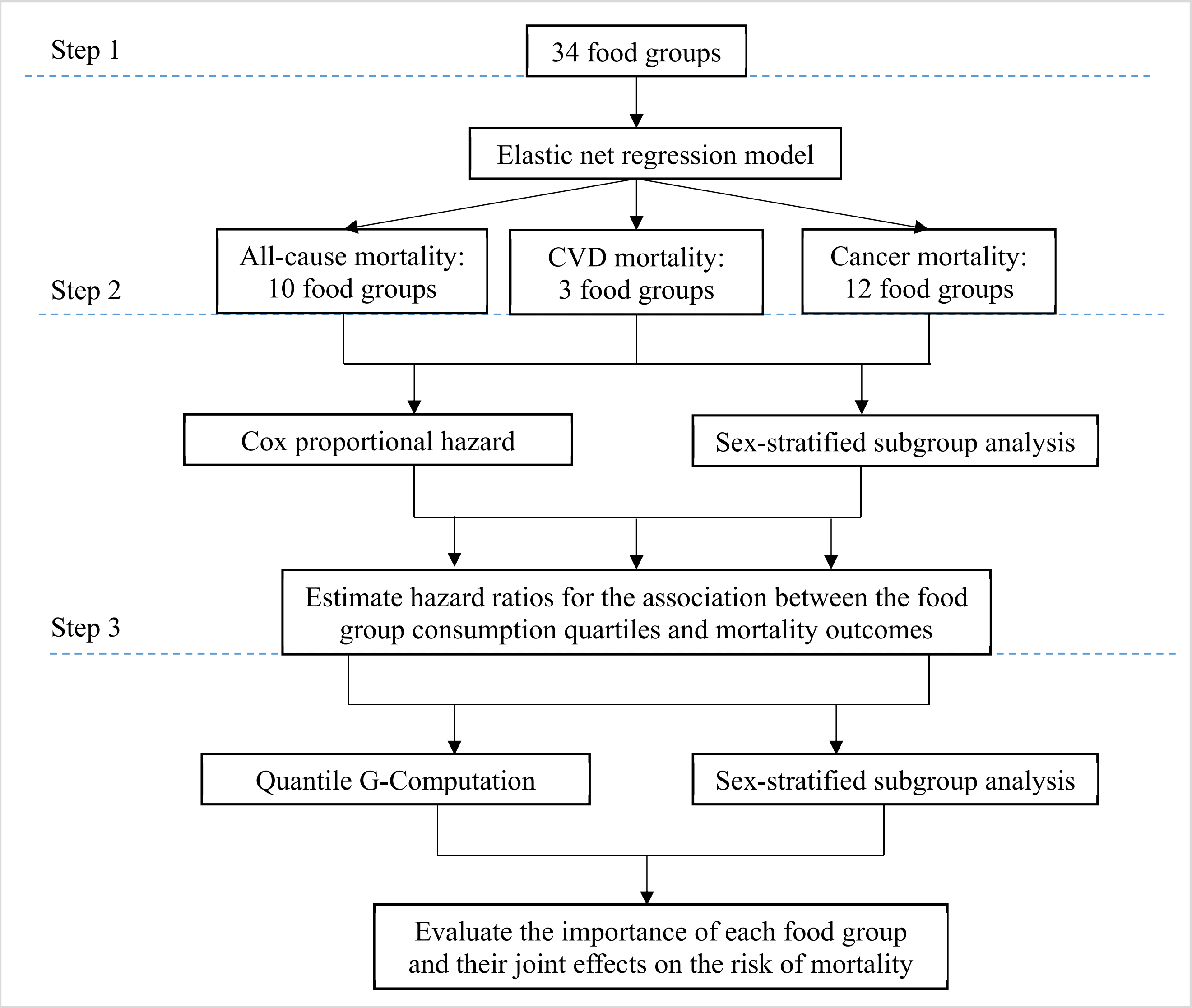

Figure S1. The chart of analytic study design

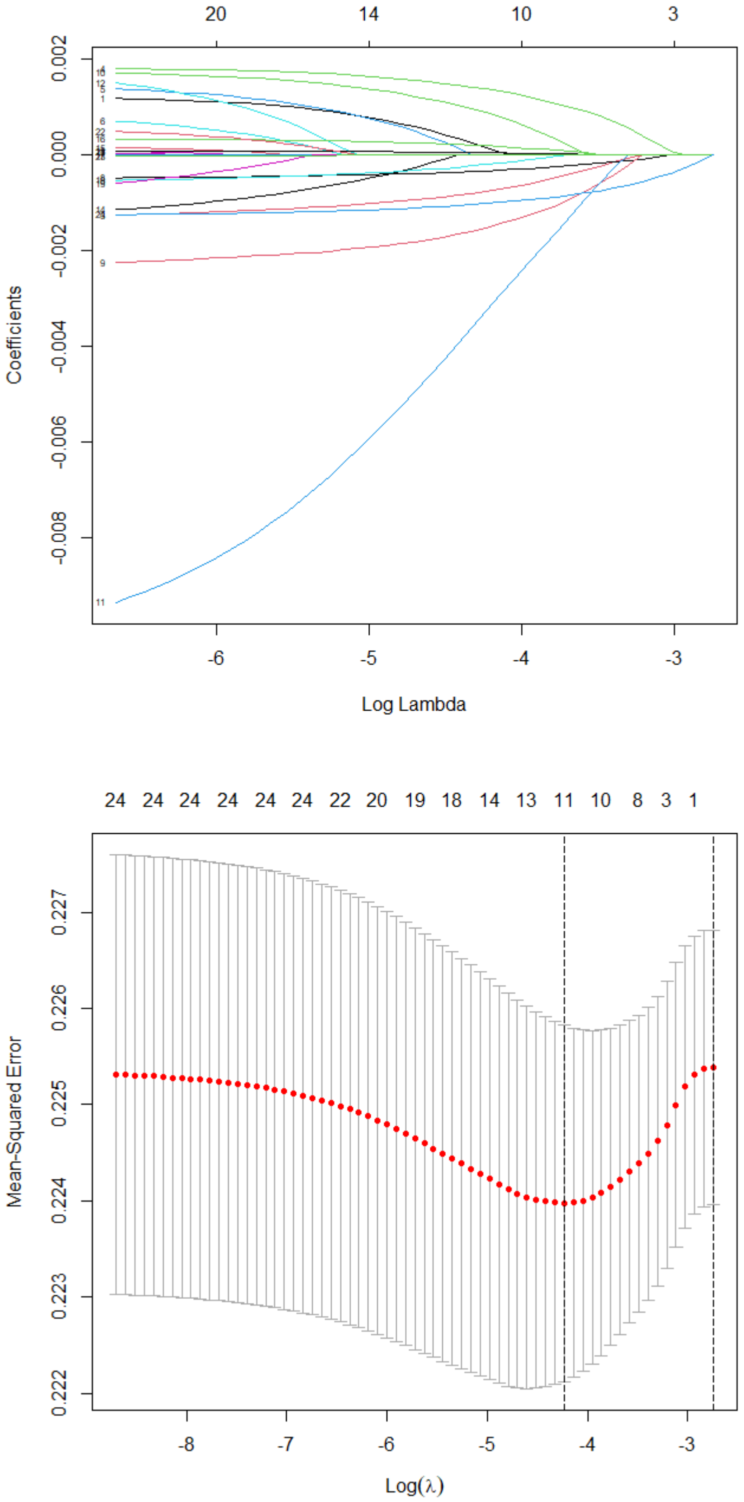

Figure S2. Elastic net analysis on the association of food groups and all-cause mortality.

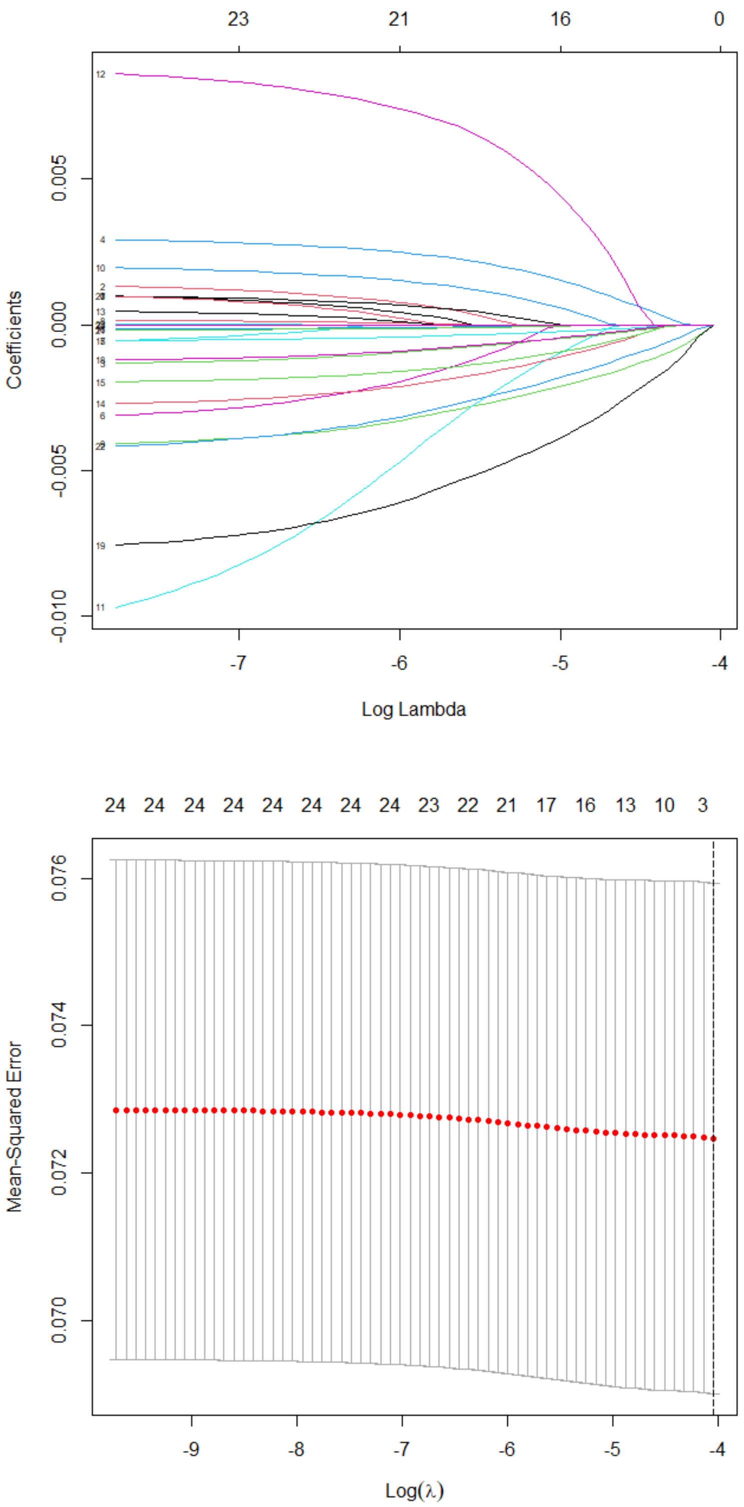

Figure S3. Elastic net analysis on the association of food groups and CVD mortality.

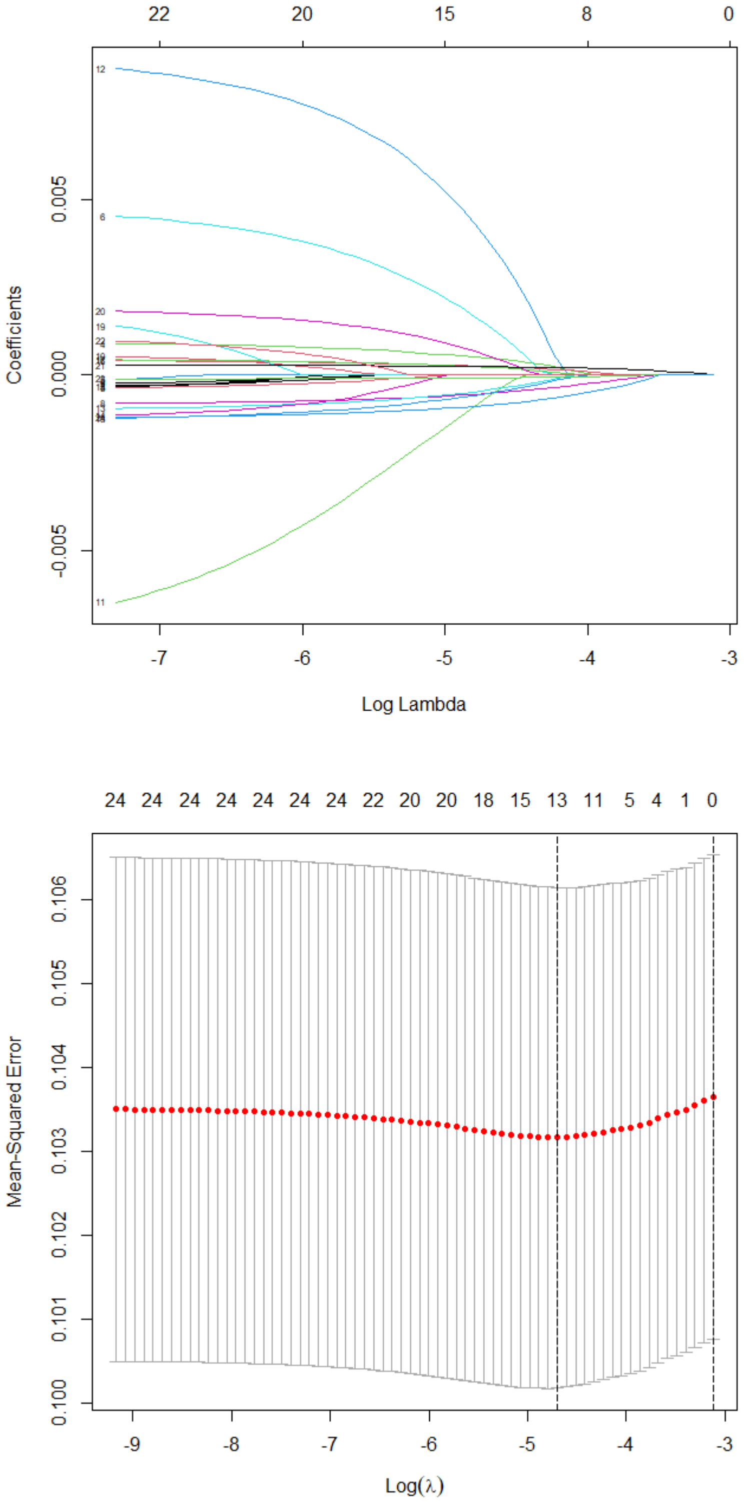

Figure S4. Elastic net analysis on the association of food groups and cancer mortality
